# Effect of Bacteria Content in Wheat Flour on Storage Stability of Fresh Wet Noodles

**DOI:** 10.3390/foods11193093

**Published:** 2022-10-05

**Authors:** Wen Yang, Kexue Zhu, Xiaona Guo

**Affiliations:** State Key Laboratory of Food Science and Technology, Jiangnan University, 1800 Lihu Avenue, Wuxi 214122, China

**Keywords:** microbial concentration, wheat flour, fresh wet noodles, storage stability

## Abstract

The effect of bacteria content in wheat flour on shelf life and storage stability of fresh wet noodles (FWNs) was evaluated in this study. Nine kinds of wheat flour with different bacterial contents were selected to make FWNs. With the increase in total plate count (TPC) from 120 CFU/g to 5500 CFU/g in flour, the shelf life of FWNs decreased from 23 d to 9 d at 4 °C. During storage, the acidity increased, which was significantly correlated with the change of TPC (*p* < 0.05), and the pH value and L* value of FWNs decreased significantly (*p* < 0.05). Changes in viscosity characteristics of starch components were also detected, the higher the TPC in flour, the more obvious the viscosity decreased. Moreover, sodium dodecyl sulfate polyacrylamide gel electrophoresis (SDS-PAGE) showed that with the deterioration of FWNs, some low molecular weight protein subunits increased; texture analysis showed that the hardness of noodles increased firstly and then decreased, the adhesiveness increased and the springiness decreased during storage. In summary, choosing flour with low TPC to prepare FWNs can extend the shelf life and slow down the quality deterioration of FWNs during storage at 4 °C.

## 1. Introduction

As a traditional staple food in China and other Asian countries, wheat-based noodles are deeply loved by consumers [1]. Fresh wet noodles (FWNs) are more popular with people because they are fresh, chewy and have a better flavor [2]. However, due to the high moisture content and rich nutrients of FWNs, microorganisms tend to increase rapidly during storage, resulting in short shelf life and great waste to enterprises [1,3]. 

At room temperature, the shelf life of FWNs is usually about 1 day [1,4]. Although the shelf life is greatly prolonged when the noodles are stored at 4 °C, the shelf life can’t meet the needs of customers [5], which hinders the industrial production of fresh noodles and severely restricts the development of this product [1,4,6]. Therefore, it is very important to reduce the initial number of microorganisms or inhibit the growth of microorganisms during the production, storage and transportation of fresh noodles [7].

In recent years, many researches are devoted to extending the shelf life of FWNs, mainly including the treatment of flour and FWNs, the use of dough mixing water with antibacterial activity and the addition of preservatives. The main factor leading to the spoilage of FWNs was the initial total plate count (TPC) in raw materials [8], superheated steam [6], ozone gas [8], electron beam irradiation [7] and heat treatment [9] were applied to wheat flour to decrease initial TPC. In terms of dough mixing water, there is mainly slightly acidic electrolyzed water [10], ozone water [11] and plasma activated water [12], these kinds of water have antibacterial activity, which can reduce the initial TPC or slow down the proliferation rate of microorganisms of FWNs during storage. Chemical or natural preservatives were added to fresh noodles to extend the shelf life of FWNs, it mainly included various chemical preservatives, such as calcium propionate and sodium dehydroacetate [1], acidity regulators, such as phosphoric acid, monosodium fumarate and fumaric acid [5] and natural antibacterial components, such as glycinin basic polypeptide and Maillard reaction products of chitosan and xylose [13,14]. Some studies also found that the treatment of produced FWNs was also effective, such as humidity-controlled dehydration treatment [15], heat treatment [16], microwave and pulsed-UV treatment [17]. However, the heat treatment of flour may affect its applicability in noodle making and the large-scale application of natural preservatives is limited due to its high cost [5]. Chemical preservatives may not completely kill microorganisms in fresh noodles and cause a certain type of microorganisms to dominate, adding excessive additives may affect the quality of noodles or produce an unpleasant smell [1]. Furthermore, now consumers are paying more and more attention to the safety of food additives, and are more interested in pure natural foods without additives [18]. Therefore, wheat flour is one of the most important factors affecting the shelf life of fresh noodles without any treatment or additives.

In general, FWNs are composed of three basic ingredients: flour, water and salt [19]. Flour accounts for the largest proportion in this formula, and the number of microorganisms in flour among the three raw materials is generally the largest. Therefore, the microbial concentration in wheat flour was one of the important factors leading to the spoilage of FWNs [8]. However, few published studies have studied in detail the influence of wheat flour with a different microbial concentration on the shelf life and the quality changes of fresh noodles during storage. Under this background, nine kinds of wheat flour with different TPC were used to prepare FWNs. These wheat flours were obtained directly from the flour mills and fresh noodles were then prepared and stored at 4 °C. During storage of FWNs, it was studied: (i) the changes in microbial concentration, pH value, acidity and brightness of FWNs, and (ii) changes in texture of cooked FWNs, and (iii) changes in starch and protein components. It may provide some reference for enterprises to select flour raw materials for making fresh noodles.

## 2. Materials and Methods

### 2.1. Materials

Nine kinds of refined flour without any treatment were obtained from five different flour mills: Wudeli Flour Mill Group Co., Ltd. (Handan, Hebei, China), COFCO Flour Industry Co., Ltd. (Haining, Zhejiang, China), Jieyang Yongxing Flour Co., Ltd. (Jieyang, Guangdong, China), Yihai Kerry Food Industry Co., Ltd. (Kunshan, Jiangsu, China) and Suiping Kemen Noodle Manufacturing Co., Ltd. (Zhumadian, Henan, China). The moisture, ash (dry basis), and crude protein content (dry basis) of wheat flour were analyzed by the AACC method 44-01.01, 08-12.01 and 39-11.01, respectively (AACC, 2000) [20]. All chemicals and reagents used in this study were of analytical grade.

### 2.2. Preparation and Storage of FWNs

The FWNs formula consisted of wheat flour and sterile water in the ratio of 100:32 (*w*/*w*). 200 g flour and 64 g water were put into a mixer, and then mixed at 95 rpm for 5 min to form uniform dough crumbles (Model JHMZ-200, Beijing, China), and then rested in sterilized plastic bags for 30 min (25 °C, 75% RH). After that, the dough crumbles were pressed into dough sheets by a noodle machine (Model JMTD-168/140, Beijing, China) 10 times. After rolling and cutting, the final fresh noodles were 20 cm in length, 1 mm in thickness and 2 mm in width. Finally, the obtained FWNs were packaged in sterilized plastic bags (25 g/bag) and stored in a refrigerator at 4 ± 1 °C. Prior to preparing FWNs, all the experimental equipment was wiped with 75% edible alcohol and irradiated with ultraviolet light for 30 min.

### 2.3. Microbial Analysis of Wheat Flour and FWNs

The determination of TPC and mold and yeast count (MYC) of flour/FWNs is referred to as GB/T 4789.2-2016 [21] and GB/T 4789.15-2016 [22], respectively. Wheat flour/FWNs (25 g) and 0.85% sterile physiological salt solution (225 mL) were put into sterile plastic bags under sterile conditions, and then slapped for 5 min with a flapping homogenizer (HX-4GM, Shanghai, China). The obtained homogenate was diluted with 0.85% sterile normal saline to obtain a series of 10-fold dilutions, and the appropriate 2–3 gradients were selected for microbial culture and counting. TPC was cultured at 36 °C for 2 days, MYC was cultured at 28 °C for 5 days, and then the plates without diffusion and with the proper number of colonies were counted. The plates with 30−300 CFU colonies were selected for TPC count, and the plates with 15−150 CFU colonies were selected for MYC count.

### 2.4. Color Measurement of FWNs Sheets

The FWNs sheets were cut into small pieces with a diameter of about 10 cm and placed in sterile bags at 4 °C. A portable colorimeter (Konica Minolta CR-400; Tokyo, Japan) was used to measure the color changes of FWNs sheets during storage using CIE L*, a* and b* color scales [23].

### 2.5. Determination of pH Value and Acidity of FWNs

The pH value of FWNs was measured with a pH meter (STARTER 3100/F, Ohaus Instruments Co., Ltd., Shanghai, China) according to the AACC method 02-52.01 (AACC, 2000) [20]. FWNs sample (10 g) was put into 100 mL of double distilled water and homogenized for 1 min to get the uniform mixture. The uniform mixture was allowed to stand for 10 min, and the pH value was immediately measured.

The determination of acidity referred to the method of Ren et al. [24] with some modifications. Double distilled water (100 mL) were added to 10 g FWNs and then homogenized for 1 min. The mixture was stirred on a magnetic stirrer for 70 min, then the suspension was allowed to stand for 30 min. Double distilled water (10 mL) and 3 drops of phenolphthalein were added to 10 mL of supernatant, then titrated with 0.01 M NaOH solution until the solution was reddish. The same volume of double distilled water was treated as a reagent blank.

### 2.6. Textural Profile Analysis (TPA)

According to the method described by Zhao et al. [25] with some modifications, a TA-XT 2i Texture analyzer (Stable Micro Systems, London, UK) was used to measure the texture characteristics of the cooked FWNs. The FWNs were initially cooked at the optimal cooking time and immediately cooled with tap water for 10 s. Each time, three strands of cooked FWNs were placed on the texture analyzer platform and compressed by a P36/R probe. The testing parameters were mode, TPA; pre-test, 1.0 mm/s; test speed, 1.0 mm/s; post-test speed, 1.0 mm/s; strain, 75%; trigger force, 5.0 g. The measurements were conducted 8 times.

### 2.7. Pasting Property Analysis

The FWNs were freeze-dried at the corresponding storage time and then ground the lyophilized FWNs into powder and passed through an 80 mesh sieve for pasting properties analysis. The pasting characteristics of the samples were determined by a rapid viscosity analyzer (RVA 4500, Perten, Macquarie Park, Australia) according to method 76-21.01 (AACC, 2000) [20].

### 2.8. Sodium Dodecyl Sulfate Polyacrylamide Gel Electrophoresis (SDS-PAGE) Analysis

SDS-PAGE analysis referred to the method of Laemmli [26] with minor modifications, 12% separation gel (pH 8.8) and 4% stacking gel (pH 6.8) were used for electrophoresis analysis. FWNs powder (40 mg) was solubilized in 1 mL of 0.01 M Tris-HCl buffer (pH 6.8), containing 10% (*w*/*v*) SDS, 0.1% (*w*/*v*) bromophenol blue, 10% (*v*/*v*) glycerol and 5% (*v*/*v*) β-mercaptoethanol. And then it was heated for 5 min in boiling water. The resulting solution was cooled to room temperature and centrifuged at 6500 g for 10 min. The supernatant (10 μL) was injected into the sample well of the gel plate. Then the electrophoresis was performed at 100 V and 75 mA during the run. Finally, the gel was stained with 0.25% *w*/*v* coomassie brilliant blue R-250, 25% absolute alcohol and 10% acetic acid and de-stained in 10% acetic acid and 25% absolute alcohol.

### 2.9. Statistical Analysis

There were three independent replicates in each experiment and the results were conducted by SPSS 25.0 (SPSS Inc.; Chicago, IL, USA) with one-way analysis of variance (ANOVA) and Duncan’s multiple range test, *p* < 0.05 showed that there were significant differences.

## 3. Result and Discussion

### 3.1. Effect of Bacteria Content in Flour on Shelf Life of FWNs1-FWNs9

#### 3.1.1. Analysis of Wheat Flour Characteristics

High-quality FWNs should have bright color, adequate shelf life and appropriate flavor and texture characteristics, and wheat flour plays a key role in all aspects of noodle quality [27]. The physicochemical properties and microbial concentration of selected flour were shown in Table 1, F1–F9 were the numbers of nine kinds of flour. A low ash level is necessary to make FWNs with a clean and white outward appearance, the selected flour had low ash content (0.40−0.55%, dry basis); an appropriate protein content is essential for the texture characteristics of FWNs, and the selected flour had moderate protein content (11−13.5%, dry basis). FWNs are prone to rapid spoilage, mainly due to the microorganisms, which adversely affect their shelf life. However, the initial number of microorganisms was one of the key factors affecting the spoilage of FWNs, which was closely related to the raw flour [8]. Therefore, nine kinds of flour were selected according to the difference in TPC in this study. Microbial concentration in F1, F2, F3 and F4 was low, the TPC was less than 300 CFU/g; microbial concentration in F5 and F6 was moderate, the TPC was about 1000 CFU/g; microbial concentration in F7, F8 and F9 was high, the TPC was more than 2000 CFU/g. The TPC of flour was generally higher than that of MYC (except for F1 and F4).

#### 3.1.2. Microbial Growth in FWNs1-FWNs9 during Storage

**Figure 1:**
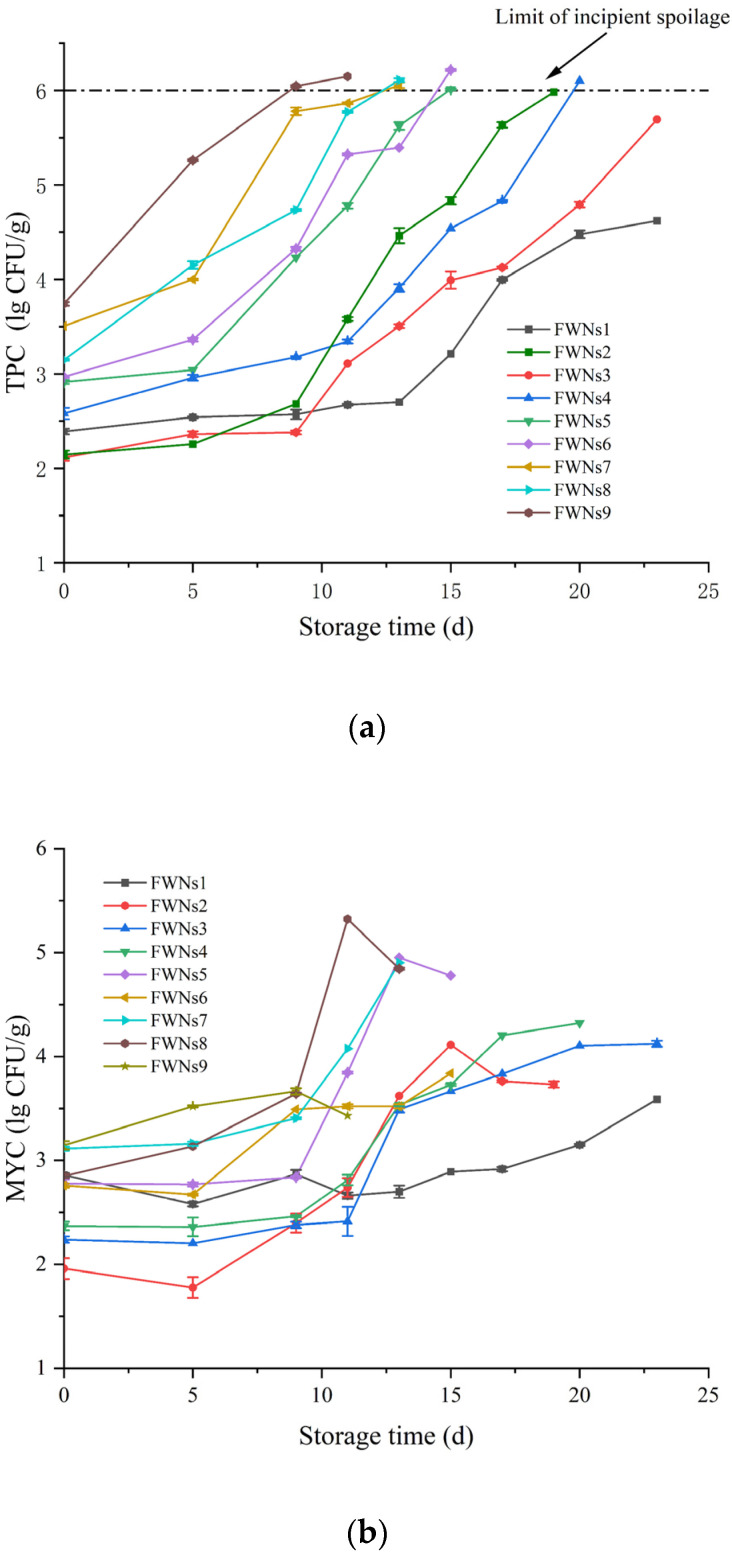
Changes of microbial concentration in FWNs prepared from flour with different bacterial concentration during storage at 4 °C. FWNs1−FWNs9 samples were prepared from F1−F9 respectively. (**a**) TPC changes in FWNs1−FWNs9 samples during storage at 4 °C; (**b**) MYC changes in FWNs1−FWNs9 samples during storage at 4 °C.

FWNs samples were prepared from nine kinds of wheat flour (F1−F9), respectively, and named as FWNs1−FWNs9. In this study, the water used for FWNs preparation was sterile and the equipment used in the whole preparation process was sterilized. Thus, the initial microbial concentration of FWNs was mainly affected by wheat flour. Figure 1a and b showed the changes of TPC and MYC of FWNs1−FWNs9 prepared from F1−F9 during storage at 4 °C. As can be seen from Figure 1a, with the increase of TPC of raw flour, the initial TPC of FWNs increased, the initial TPC in FWNs was similar to that in flour, and some increased or decreased might be due to the rest process of dough crumbles or the rolling process. Moreover, the colony morphology of freshly prepared FWNs was highly consistent with that of flour (picture not shown), the results were consistent with the description of Li et al. [8] that the main source of bacteria in FWNs was raw materials.

Generally speaking, the rapid deterioration of processed food is caused by bacteria, followed by yeast and mold. Therefore, TPC was usually used as an important indicator of fresh noodle spoilage [5,28]. The cut-off point of FWNs between spoiled and unspoiled (limit of incipient spoilage) was 10^6^ CFU/g according to the literature [28,29] or mildew spots appeared on the surface of noodles [24]. As shown in Figure 1a, the TPC of all samples showed an upward trend during storage, but the time when TPC began to rise rapidly was different. TPC of FWNs1, FWNs2, FWNs3 and FWNs4 was basically unchanged after storage for 9 days, and rapidly increased after the 9th day, reached the limit of incipient spoilage around the 20th day; TPC of FWNs5 and FWNs6 was basically unchanged after storage for 5 days, and rapidly increased after the 5th day, reached the limit of incipient spoilage around the 15th day; TPC of FWNs7, FWNs8 and FWNs9 increased rapidly in the early stage of storage, and reached the limit of incipient spoilage around the 10th day. TPC in FWNs1, FWNs2, FWNs3 and FWNs4 increased slowly at the early stage of storage, which might be related to the low concentration of bacteria in flour or some mesophilic bacteria couldn’t adapt to the storage environment of 4 °C immediately [3]. While FWNs7, FWNs8 and FWNs9 increased rapidly in the early stage of storage, this might be due to the high initial bacterial concentration.

As shown in Figure 1b, the MYC of all samples showed an upward trend during storage, except for the decrease in later periods. Microbial spoilage was a complex process [30], and the interaction of microorganisms in this process might inhibit or accelerate their spoilage activity [31]. The growth trend of MYC in FWNs was not synchronized with bacteria, and began to increase rapidly after the ninth day, which might be due to the fact that the initial MYC in FWNs was lower than that of TPC.

Under the storage condition of 4 °C, bacteria were the primary factor causing spoilage of FWNs and no visible mildew spots appeared during this period. The shelf life of FWNs decreased significantly (*p* < 0.05) with the increase of TPC in raw flour and it was significantly correlated with the TPC in flour (*p* < 0.01). With the increase of TPC from 120 CFU/g to 5500 CFU/g in wheat flour, the shelf life of FWNs decreased from 23 d to 9 d at 4 °C. Huang et al. [6] treated the raw flour with superheated steam, the TPC decreased by 1.23 lg CFU/g, and the shelf life of fresh noodles was extended to two days at 25 °C. Similarly, Hong et al. [9] prepared fresh noodles with heat-treated flour, with the increase in heat-treated temperature, the initial microbial concentration of FWNs decreased and the shelf life of FWNs was prolonged. These results showed that the high bacterial concentration of raw wheat flour was the main reason for the high initial bacterial concentration and short shelf life of FWNs. Thus, it is an effective way to prolong the shelf life of fresh noodles by reducing the concentration of bacteria in raw flour. Flour production mainly includes cleaning, tempering, and grinding. Therefore, it is possible to strengthen the cleaning of the wheat grain surface, control the increase of microorganisms in the tempering process, clean processing or treat wheat flour directly to produce wheat flour with low bacterial concentration, so as to extend the shelf life of fresh noodles.

### 3.2. pH Value and Acidity Changes of FWNs1–FWNs9

**Figure 2:**
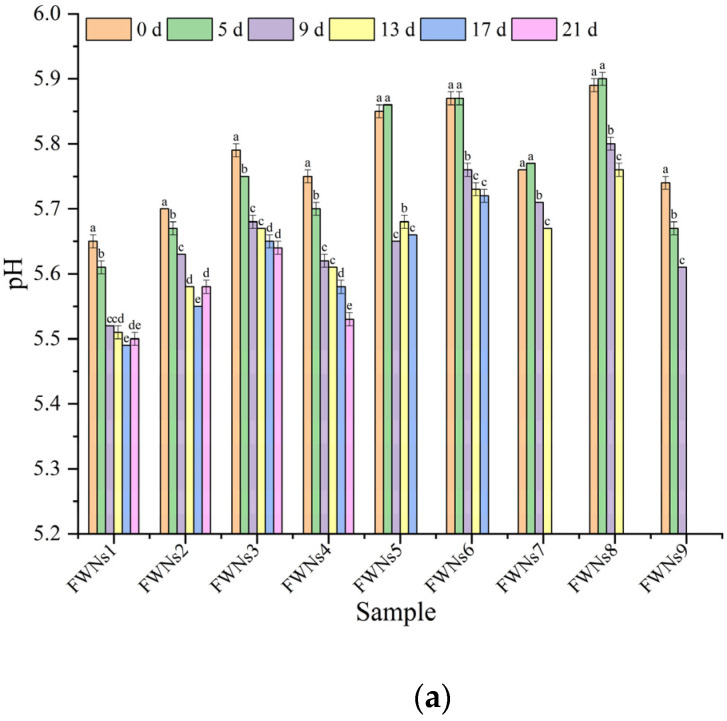

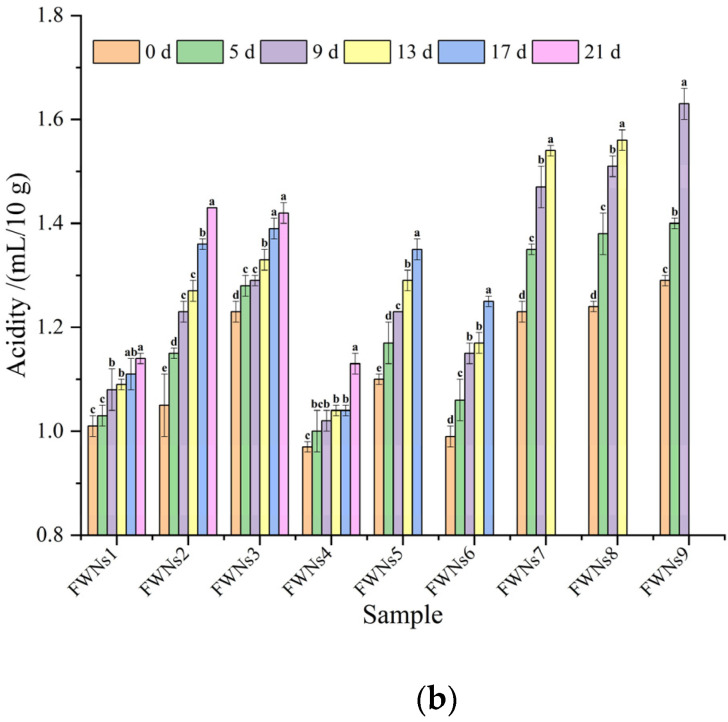
Changes of pH value (**a**) and acidity (**b**) in FWNs1−FWNs9 samples during storage at 4 °C. Different letters in the same group represent significant differences (*p* < 0.05).

The changes in pH value of FWNs during storage at 4 °C were shown in Figure 2a. The initial pH values of FWNs prepared from different flour were different, which might be related to the properties of flour. During storage, the pH value of all samples showed a downward trend and decreased significantly (*p* < 0.05). The initial pH value of FWNs1, FWNs2 and FWNs3 was low, and the pH value decreased continuously during the whole storage period. The pH value of FWNs1, FWNs2 and FWNs3 decreased by 0.15, 0.12 and 0.15 from the beginning to the 21st day, respectively, and the degree of decline was basically the same. In the first nine days of storage, the pH value of FWNs1, FWNs2 and FWNs3 decreased greatly, and it was relatively gentle in the later period of storage. The initial pH of FWNs5, FWNs6 and FWNs8 was relatively high, and the pH basically remained unchanged in the first five days, and then decreased significantly after five days of storage. As can be seen from Figure 1a, bacteria also began to increase rapidly after five days. The pH value of all samples decreased by about 0.15−0.20 from the beginning to the end of storage. It was common that the pH value of FWNs to decrease due to the growth and proliferation of microorganisms. FWNs were rich in carbohydrates and protein, these nutrients were beneficial to microbial growth, and microbes metabolized acids and led to the decrease of pH value [28]. Studies by Wu et al. [32] showed that the amount of acid in fresh noodles increased sharply during storage. Li et al. [23] kept fresh noodles at room temperature for 3 days, and the pH value of FWNs decreased from 6.18 to 5.70.

In general, the pH value decrease of FWNs was consistent with the change of TPC, which indicated that fermentation caused by microbial reproduction was a spoilage type for FWNs [33]. Moreover, it was found that the pH value was stable or even slightly increased in the late storage period, which might be related to the decomposition of protein in FWNs and the release of some basic compounds such as amine and ammonia [23].

In addition, Figure 2b showed the changes in the acidity of FWNs during storage at 4 °C. The acidity of FWNs in different groups increased to different degrees during storage. As shown in Figure 2b, the acidity of FWNs1 increased from 1.01 to 1.14 and that of FWNs2 increased from 1.05 to 1.43 during storage. However, the initial microbial concentration of the two groups of FWNs was about the same, which might be due to the fact that the microbial growth rate of FWNs2 was much higher than that of FWNs1 during storage (as shown in Figure 1a). During storage, microorganisms constantly grew and reproduced, and the nutrients (carbohydrates and lipids) in FWNs were metabolized by microorganisms into various organic acids, such as citric acid and lactic acid, which resulted in an increased acidity [34]. The acidity changes of FWNs5 and FWNs6 were basically the same during storage, which might be related to the same initial microbial concentration and microbial changes during storage; the acidity of FWNs7 and FWNs8 changed greatly, which might be related to the higher initial TPC or the faster microbial growth during storage. Besides, the change of acidity of all samples during storage was significantly related to the change of TPC (*p* < 0.05), which was consistent with the conclusion of Xiong et al. [16]. Therefore, controlling the initial microbial concentration and the proliferation during storage was very beneficial to reduce the acidity increase of fresh noodles during storage.

### 3.3. Color Changes of FWNs1−FWNs9

**Figure 3:**
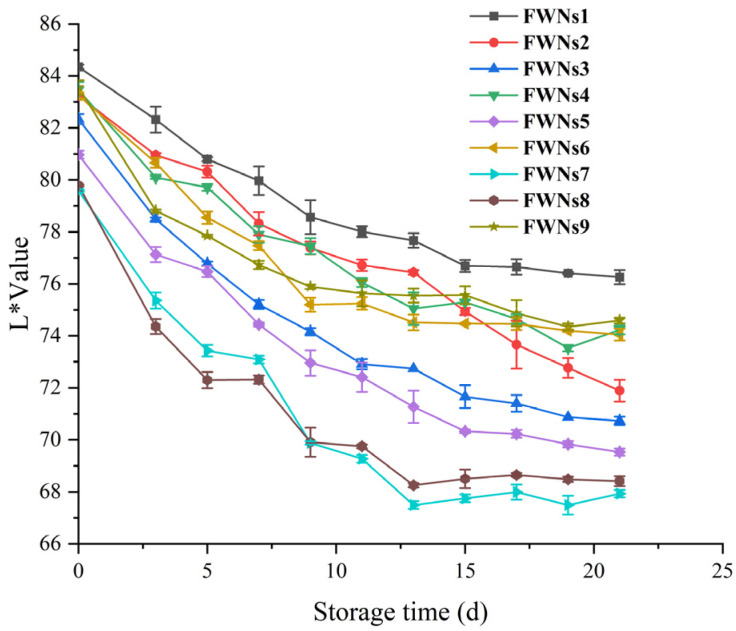
L* value changes of FWNs1−FWNs9 during storage at 4 °C.

Color plays an important role in affecting the marketability of FWNs [27]. Typically, freshly prepared FWNs are bright white, and they tend to darken easily during storage, which reduces the marketability of FWNs [27,35]. The changes in the L* value of nine groups of FWNs were shown in Figure 3. There were differences in the initial L* value of different groups, the initial L* value of FWNs1 was the highest, and FWNs8 was the lowest, which were 83.5 and 79.6, respectively. These differences might be related to the processing accuracy of different raw flours. The ash and protein content of F1 were lower, which were 11.01% and 0.41% respectively, and those of F8 were 12.15% and 0.54% respectively (shown in Table 1). Generally speaking, low flour extraction and ash levels are preferred for the manufacture of noodles with a clean and bright appearance. In addition, the increase of protein content in flour reduces the L* value of FWNs [27,36]. Starch is the most abundant constituent in wheat flour, the content of starch and the ratio of amylose/amylopectin also affect the initial L* value of FWNs. According to the study of Hu et al. [37], the L* value of fresh noodles was negatively correlated with the ratio of amylose/amylopectin in wheat flour.

The L* values of all samples showed a downward trend during the whole storage. The value of L* decreased rapidly in the early stage (0−5 d). After storage at 4 °C for 5 days, the L* value of FWNs1 decreased by 3.8, while that of FWNs8 decreased by 7.3, which might be related to the high ash content of F8. Previous studies showed that polyphenol oxidase (PPO) enzyme activity was an important factor affecting the browning of fresh noodles [38]. PPO enzyme could interact with polyphenols in noodles to produce brown substances [39]. With the increase in flour yield, the ash content of flour increases, and the PPO enzyme activity and polyphenol content in fresh noodles are higher [40], resulting in browning. The L* value decreased slowly in the later stage, which might be due to the consumption of browning substrate polyphenols [23].

The browning of FWNs sheets is mainly related to PPO enzyme activity, wheat varieties and protein content in flour [41]. The reduction streams with low ash content and high processing accuracy can be blended into special flour for fresh noodles [40], or choose wheat varieties with low PPO enzyme activity to process flour [38].

### 3.4. Texture Changes of FWNs1, FWNs6 and FWNs7

The texture is a key quality determinant of FWNs [42]. In order to study the effects of different microbial concentrations in flour on the texture changes of FWNs during storage, F1, F6 and F7 were selected to prepare fresh noodles, respectively. F1 had a low microbial concentration, F6 had a medium microbial concentration and F7 had a relatively high microbial concentration. The texture changes of FWNs1, FWNs6 and FWNs7 during storage were measured, including hardness, adhesiveness, springiness and chewiness. The data were shown in Table 2.

The initial texture properties of FWNs1, FWNs6 and FWNs7 were different due to different flour materials. During storage at 4 °C, the hardness of FWNs increased in the early stage, which might be due to water migration and loss, it was difficult for water to enter the center of FWNs when cooking. Another reason might be the further development of the gluten network during early storage [23]. The hardness of FWNs decreased during the later storage period, the hardness of FWNs7 began to decrease after 9 days of storage, and FWNs1 and FWNs6 began to decrease after 13 days of storage. The hardness of FWNs7 decreased earlier than that of FWNs1 and FWNs6, which might be related to the fact that FWNs7 had a high initial microbial concentration, which caused great damage to FWNs texture [43]. In addition, with the prolongation of storage time, the acidity of FWNs increased and the pH decreased, which might damage the ingredients in the FWNs matrix and lead to a decrease in hardness [5].

The adhesiveness of FWNs1, FWNs6 and FWNs7 increased by 25%, 42% and 101% respectively during whole storage. Along with the extension of storage time, the growth and metabolism of microorganisms led to the migration of water in the noodles to the surface [23] and destroyed the protein network, the hydration capacity of protein decreased and surface soluble substances increased after cooking, resulting in the adhesiveness of FWNs increased after cooking [6,44]. There was no significant change in the springiness of FWNs1 and FWNs6 during storage at 4 °C (*p* > 0.05), FWNs7 decreased significantly in the later storage period (*p* < 0.05), this might be due to the fact that the initial bacterial amount and the microbial growth rate during storage of FWNs7 were higher than FWNs1 and FWNs6. Enzymes metabolized by microorganisms might cause protein degradation and destroy the protein network, which might lead to a decrease in springiness [45,46]. Zhang et al. [2] reported the gluten network collapsed obviously and the GMP weight decreased significantly after storage at 25 °C for two days, these changes may be due to microbial activity or other deterioration.

There was the same trend of texture deterioration among the three groups of FWNs, but the deterioration rate of FWNs was relatively slow due to low storage temperature, low initial microbial concentration, slow microbial proliferation and low enzymatic activity under refrigeration conditions.

### 3.5. Changes of the Starch Components in FWNs1, FWNs6 and FWNs7

**Figure 4:**
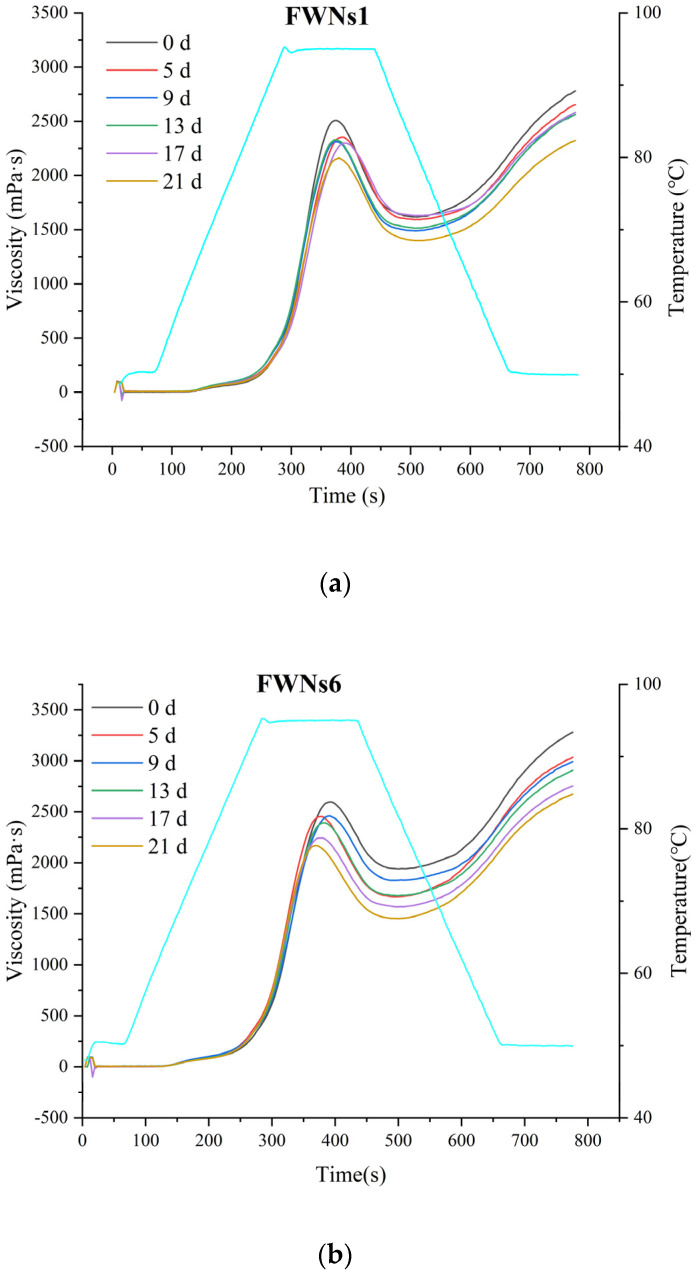

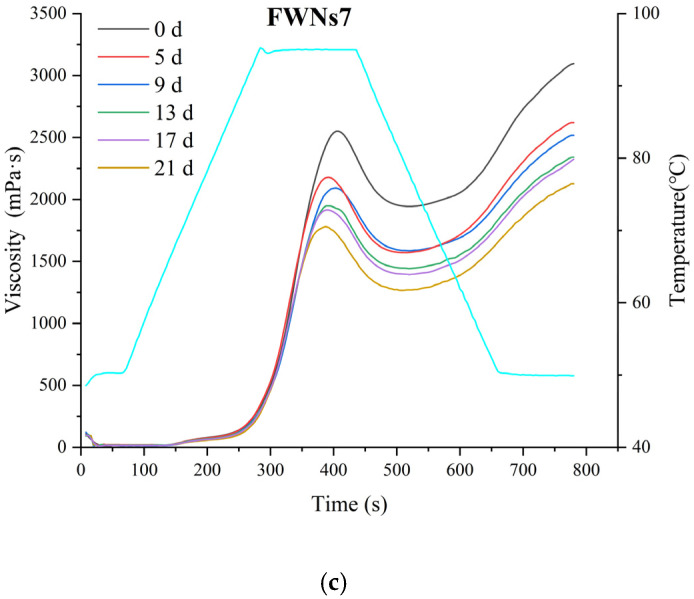
Changes of pasting curves of FWNs1, FWNs6 and FWNs7 during storage at 4 °C. (**a**) Changes of pasting curves of FWNs1 prepared from F1 during storage; (**b**) Changes of pasting curves of FWNs6 prepared from F6 during storage; (**c**) Changes of pasting curves of FWNs7 prepared from F7 during storage.

The pasting property of starch is closely related to the quality characteristics of noodles [47]. The viscosity changes of starch components of FWNs1, FWNs6 and FWNs7 during storage were summarized in Figure 4. The peak viscosity, trough viscosity and final viscosity of starch components of three groups of FWNs showed a downward trend with the extension of storage time. After five days of storage, the peak viscosity of FWNs1, FWNs6 and FWNs7 decreased by 155, 145 and 356 mPa·s respectively, but the microbial concentration changes were small (Figure 1a,b), which might be related to the activation of amylase during the preparation of FWNs [23].

In the whole storage process, the peak viscosity of FWNs1, 6 and 7 decreased from 2507, 2598 and 2554 mPa·s to 2161, 2244 and 1781 mPa·s, respectively. The viscosity of starch components decreased at a rate of FWNs7 > FWNs6 > FWNs1, which was consistent with the number and growth rate of microorganisms in FWNs1, FWNs6 and FWNs7. Microbial metabolites might cause the decomposition of starch molecules [17]. In addition, the dominant bacteria in wheat flour are mainly *Bacillus* spp. and *Micrococcus* spp. [1], which may produce amylase during the growth and reproduction, leading to partial decomposition of starch and loss of its thermal gelatinization and viscosity characteristics [48]. On the other hand, during storage, the acidity of noodles increased and the pH value decreased, and the interaction between microbial metabolites and starch might lead to the change in starch viscosity characteristics. According to the research of Han et al. [5], with the decrease in pH value of FWNs, the peak viscosity and final viscosity decreased. Studies by Sriburi et al. [49] showed that low pH values might induce starch hydrolysis. The viscosity characteristics of starch components changed might due to microbial growth, metabolism and biochemical reaction [23].

With the increase of the microbial concentration in flour, the growth rate of microorganisms in FWNs during storage increased and the decrease rate of starch viscosity accelerated. Therefore, reducing the microbial concentration in flour may improve the quality stability of FWNs during storage.

### 3.6. SDS-PAGE Analysis of FWNs1, FWNs6 and FWNs7

**Figure 5:**
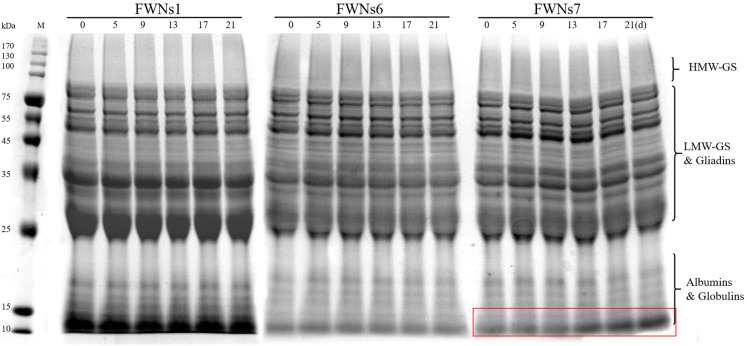
Protein subunits changes of FWNs1, FWNs6 and FWNs7 during storage at 4 °C. The intensity of the bands in the low molecular weight region increased and it was highlighted with a red rectangle.

The protein component is one of the key factors to determine the quality of fresh noodles. Therefore, the changes in protein subunits of FWNs during storage were observed by SDS-PAGE. It can be seen from Figure 5, the protein subunits were mainly divided into three components according to electrophoretic mobility, including high-molecular-weight glutenin subunits (HMW-GS), low-molecular-weight glutenin subunits (LMW-GS) and gliadin, albumin and globulin [50]. Moreover, the position of the subunit bands of the three groups of FWNs was basically the same, but the color depth of the bands was different, which might be related to the different protein subunit ratios of different flour materials.

It can be seen from Figure 5 that almost no bands disappeared or new bands appeared during storage of FWNs1, FWNs6 and FWNs7. With the extension of storage time, the electrophoresis profiles of FWNs1 and FWNs6 remained basically unchanged, while the intensity of the bands in the low molecular weight region of FWNs7 increased (Figure 5, highlighted with red rectangle). It might be due to the decomposition of some high molecular weight proteins, resulting in the increase of low molecular weight subunits. During storage, the microbial growth rate of FWNs7 was faster than that of FWNs1 and FWNs6. The growth, reproduction and metabolism of microorganisms may produce some hydrolases, which lead to the decomposition of some protein components in FWNs. In addition, microorganisms themselves might also produce low-molecular-mass nitrogen metabolites [51]. Similarly, the springiness of FWNs7 decreased during storage, while the FWNs1 and FWNs6 remained basically unchanged. Gluten protein plays an important role in the viscoelastic properties of noodles. Some protein components might be degraded and the gluten network destroyed due to microbial proliferation and hydrolase [40], which might lead to the deterioration of FWNs7 springiness.

These results showed that the higher the microbial concentration in raw flour, the faster the microbial proliferation and the more serious the changes in protein components and destruction of the gluten network in FWNs during storage. Reducing the destruction of the gluten network is one of the important factors to maintain the quality of fresh noodles during storage [45]. Therefore, reducing the microbial concentration in raw materials can indirectly reduce the decomposition of protein components and texture deterioration of FWNs during storage.

## 4. Conclusions

The microbial concentration of wheat flour plays a key role in the storage stability of FWNs. With the increase of TPC in raw flour, the initial TPC of FWNs increased, the shelf life of FWNs was gradually shortened, and its shelf life was significantly correlated with the TPC of raw flour (*p* < 0.01). During the storage of FWNs, microbes metabolized acids, which led to an increase in acidity and a decrease in pH value. In addition, the initial L* value of FWNs made from flour with low ash and protein content was higher. With the increase of initial TPC in flour, the viscosity changes of starch component and the texture deterioration speed of FWNs increased, and the protein component of FWNs was degraded more obviously during storage.

To sum up, selecting flour with low microbial concentration to make FWNs is helpful to prolong the shelf life of FWNs and slow down their quality deterioration during storage. Therefore, when selecting flour raw materials, fresh noodles companies should consider not only the processing characteristics of flour, but also the microbial concentration. Further studies should be performed on the changes of microbial species during storage and the influence of developed bacteria on the deterioration speed of FWNs.

## Figures and Tables

**Table 1:** Physiochemical parameters and microbial concentration of nine kinds of wheat flour.

Flour Sample	Water (%)	Protein (%)	Ash (%)	TPC (CFU/g)	MYC (CFU/g)
F1	12.13 ± 0.00 ^g^	11.01 ± 0.01 ^d^	0.41 ± 0.01 ^d^	120 ± 10 ^e^	530 ± 0 ^d^
F2	12.62 ± 0.09 ^e^	13.38 ± 0.11 ^a^	0.42 ± 0.03 ^d^	120 ± 10 ^e^	130 ± 20 ^f^
F3	12.70 ± 0.01 ^e^	12.09 ± 0.02 ^c^	0.47 ± 0.02 ^c^	220 ± 20 ^e^	210 ± 10 ^f^
F4	13.74 ± 0.02 ^a^	11.34 ± 0.00 ^d^	0.43 ± 0.01 ^d^	280 ± 10 ^e^	410 ± 10 ^e^
F5	13.32 ± 0.00 ^b^	12.26 ± 0.05 ^b^	0.47 ± 0.00 ^c^	940 ± 30 ^d^	720 ± 0 ^c^
F6	12.31 ± 0.05 ^f^	12.18 ± 0.03 ^c^	0.43 ± 0.01 ^d^	990 ± 10 ^d^	580 ± 20 ^d^
F7	13.18 ± 0.03 ^c^	12.02 ± 0.01 ^c^	0.50 ± 0.00 ^b^	2400 ± 200 ^c^	720 ± 30 ^c^
F8	13.00 ± 0.04 ^d^	12.15 ± 0.01 ^c^	0.54 ± 0.01 ^a^	2600 ± 100 ^b^	900 ± 50 ^b^
F9	13.30 ± 0.06 ^b^	10.96 ± 0.00 ^d^	0.43 ± 0.00 ^d^	5500 ± 100 ^a^	1400 ± 100 ^a^

F1 to F9 are the numbers of nine kinds of flour. Different letters in the same column indicate significant differences at the *p* < 0.05 level.

**Table 2:** Texture changes of FWNs1, FWNs6 and FWNs7 during storage.

Sample	Storage Time (d)	Hardness (g)	Adhesiveness (g·s)	Springiness	Chewiness
FWNs1	0	3880 ± 146 ^d^	−88 ± 15 ^a^	0.92 ± 0.01 ^a^	2461 ± 51 ^ab^
	5	4136 ± 102 ^bcd^	−90 ± 15 ^a^	0.94 ± 0.02 ^a^	2556 ± 78 ^a^
	9	4320 ± 40 ^b^	−101 ± 11 ^ab^	0.94 ± 0.01 ^a^	2538 ± 80 ^a^
	13	4699 ± 158 ^a^	−129 ± 29 ^b^	0.92 ± 0.02 ^a^	2575 ± 75 ^a^
	17	4010 ± 225 ^cd^	−88 ± 14 ^a^	0.93 ± 0.01 ^a^	2238 ± 91 ^c^
	21	4245 ± 186 ^bc^	−110 ± 17 ^ab^	0.93 ± 0.02 ^a^	2396 ± 48 ^b^
FWNs6	0	3807 ± 100 ^c^	−93 ± 20 ^a^	0.92 ± 0.03 ^a^	2411 ± 95 ^b^
	5	4628 ± 188 ^a^	−143 ± 21 ^ab^	0.92 ± 0.01 ^a^	2643 ± 116 ^b^
	9	4560 ± 164 ^a^	−134 ± 36 ^ab^	0.92 ± 0.01 ^a^	2626 ± 151 ^a^
	13	4811 ± 111 ^a^	−164 ± 38 ^b^	0.89 ± 0.02 ^a^	2625 ± 51 ^a^
	17	4254 ± 115 ^b^	−117 ± 28 ^ab^	0.92 ± 0.03 ^a^	2396 ± 98 ^a^
	21	4294 ± 157 ^b^	−131 ± 19 ^ab^	0.91 ± 0.03 ^a^	2390 ± 134 ^b^
FWNs7	0	3579 ± 117 ^c^	−68 ± 13 ^a^	0.90 ± 0.01 ^ab^	2224 ± 40 ^c^
	5	4522 ± 145 ^ab^	−105 ± 10 ^b^	0.92 ± 0.01 ^a^	2553 ± 170 ^ab^
	9	4694 ± 66 ^a^	−119 ± 11 ^bc^	0.92 ± 0.03 ^a^	2725 ± 35 ^a^
	13	4492 ± 124 ^ab^	−142 ± 22 ^c^	0.89 ± 0.02 ^ab^	2393 ± 110 ^bc^
	17	4290 ± 251 ^b^	−106 ± 25 ^b^	0.89 ± 0.05 ^ab^	2311 ± 254 ^bc^
	21	4331 ± 237 ^b^	−135 ± 25 ^bc^	0.88 ± 0.02 ^b^	2302 ± 165 ^bc^

FWNs1, FWNs6 and FWNs7 were prepared from F1, F6 and F7, respectively. Different letters in the same column indicate significant differences at the *p* < 0.05 level.

## Data Availability

Not applicable.
